# U. S. Marine Hospital

**Published:** 1868-09-15

**Authors:** 


### *U. S. Marine Hospital at Chicago.*

The report of the accomplished special agent and medical inspector of Marine Hospitals, W. D. STEWART, M.D., on the inspection of our Marine Hospital, is very complimentary to Surgeon Rogers, who has it in charge. This was to have been expected by all who know Dr. R. The only objection to him is he *won't* get married and thus *perpetuate those like him.*

